# Effectiveness of an intervention with mothers to stimulate children under two years*

**DOI:** 10.1590/1518-8345.3176.3216

**Published:** 2019-10-28

**Authors:** Isolda Maria Barros Torquato, Neusa Collet, Franklin Delano Soares Forte, Jael Rúbia Figueiredo de Sá França, Maria de Fátima de Oliveira Coutinho Silva, Altamira Pereira da Silva Reichert

**Affiliations:** 1 Universidade Federal da Paraíba, João Pessoa, PB, Brazil.

**Keywords:** Child Development, Child Day Care Centers, Pediatric Nursing, Health Education, Mothers, Early Intervention, Desenvolvimento Infantil, Creche, Enfermagem Pediátrica, Educação em Saúde, Mães, Intervenção Precoce, Desarrollo Infantil, Guardería, Enfermería Pediátrica, Educación en Salud, Madres, Intervención Precoz

## Abstract

**Objective:**

To analyze the effectiveness of an educational intervention with mothers to stimulate children under two years of age at risk for neuropsychomotor development.

**Method:**

Before-after intervention study, conducted with 52 mothers of children under two years old, enrolled in reference centers in early childhood education. Initially, maternal knowledge regarding child development and stimulation was assessed through a structured instrument. Then, workshops were held with the mothers and, after three months, maternal knowledge was revised, reapplying the data collection instrument. In the analysis, descriptive and inferential statistics were used, applying the McNemar and Wilcoxon tests and the Rasch Model from the Item Response Theory.

**Results:**

after the intervention, there was a significant increase in scores regarding the following aspects: knowledge of mothers about child development and stimulation from 5.77 ± 1.85 to 18.60 ± 1.94 (p <0.001); reduction of the maternal difficulty index in answering the instrument questions from 1.17 ± 0.57 to -1.98 ± 1.63 (p 0.01).

**Conclusion:**

the educational intervention contributed to the improvement of maternal knowledge regarding the development and forms of child stimulation, corroborating the importance of this action to advance the health of children at risk under maternal care at home.

## Abstract (pt)

**Objetivo:**

analisar a efetividade de uma intervenção educativa com mães para a estimulação de crianças menores de dois anos em situação de risco para o desenvolvimento neuropsicomotor.

**Método:**

estudo de intervenção do tipo antes-depois, realizado com 52 mães de crianças menores de dois anos e matriculadas em centros de referências em educação infantil. Inicialmente, foram avaliados os conhecimentos maternos quanto ao desenvolvimento e à estimulação infantil por meio de instrumento estruturado. Em seguida, realizou-se oficinas com as mães e, após três meses, os conhecimentos maternos foram reavaliados, reaplicando o instrumento de coleta de dados. Na análise, utilizou-se estatística descritiva e inferencial, sendo aplicados teste de McNemar, de Wilcoxon e o modelo de Rash da Teoria da Resposta ao Item.

**Resultados:**

após intervenção, houve aumento significativo dos escores quanto aos aspectos: conhecimento das mães sobre o desenvolvimento e estimulação infantil de 5,77 ± 1,85 para 18,60 ± 1,94 (p < 0,001); e redução do índice de dificuldade materna em responder as questões do instrumento de 1,17 ± 0,57 para −1,98 ± 1,63 (p < 0,01).

**Conclusão:**

a intervenção educativa contribuiu para a melhora dos conhecimentos maternos quanto ao desenvolvimento e às formas de estimulação infantil, corroborando a importância desta ação para o avanço da saúde da criança com risco sob os cuidados maternos no domicílio.

## Abstract (es)

**Objetivo:**

analizar la efectividad de una intervención educativa con madres para la estimulación de niños menores de dos años en situación de riesgo, para el desarrollo neuropsicomotor.

**Método:**

estudio de intervención del tipo antes-después, que fue realizado con 52 madres de niños menores de dos años, inscriptos en centros de referencias en educación infantil. Inicialmente, fueron evaluados los conocimientos maternos cuanto al desarrollo y la estimulación infantil por medio de instrumento estructurado. Luego, fueron realizados talleres con las madres y, después de tres meses, los conocimientos maternos reevaluados, reaplicando el instrumento de recogida de datos. En el análisis, se utilizó estadística descriptiva e inferencial, siendo aplicadas pruebas McNemar, Wilcoxon y Modelo de Rash de la Teoría de la Respuesta al Ítem.

**Resultados:**

después de la intervención, hubo incremento significativo de los escores cuanto a los aspectos: conocimiento de las madres sobre el desarrollo y la estimulación infantil de 5,77±1,85 para 18,60±1,94 (p<0,001); reducción del índice de dificultad materna en responder las cuestiones del instrumento de 1,17 ± 0,57 para -1,98 ± 1,63 (p<0,01).

**Conclusión:**

la intervención educativa contribuyó para la mejora de los conocimientos maternos cuanto al desarrollo y a las formas de estimulación infantil, corroborando la importancia de esta acción para el avance de la salud del niño con riesgo bajo los cuidados maternos en el domicilio.

## Introduction

Child development, initiated in intrauterine life, is defined as a complex and dynamic process related to physical growth, neurological maturation, and progressive acquisition of motor and psychocognitive skills in children^(^ 1 ^)^.

Although continuous, qualitative and sequential, development may present its chronological course compromised in different domains due to the influence of risk factors, making the child more vulnerable to facing the evolutionary tasks of their life cycle^(^ 2 ^)^. Risk factors for child development are those of genetic, biological origin and those associated with poor health and housing conditions, inadequate care and education practices, and an affectively disrupted home environment (environmental risk)^(^ 3 ^)^.

Cumulative exposure to these risk factors still in childhood may reflect negatively on maturing brain function, increasing the chances of motor, cognitive, behavioral and/or language disorders, which notably affect overall development and the child’s learning process^(^ 4 ^)^.

Estimates show that 200 million children under the age of five worldwide are at risk of not achieving their developmental potential^(^ 5 ^)^. A study found that 43% of children under five (about 250 million) living in low- and middle-income countries are at risk of developmental delays that, early in life, may lead to health and learning problems and inadequate nutrition, reflecting in low wages in adulthood, as well as social tensions, with negative consequences not only for the present generation, but also for the future ones^(^ 6 ^)^.

Risk factors may also precede socioeconomic variables in adulthood, such as crime, negative influence on school performance, especially of women, and intergenerational transmission of poverty^(^ 7 ^-^ 8 ^)^. Thus, early identification of changes in neuropsychomotor development is fundamental for decision-making regarding referral to specialized treatment in a timely manner, which will reflect a greater chance of reversal of delays in children and a better future for them^(^ 9 ^)^.

In this context, the importance of child health surveillance actions is highlighted, including different programs, specifically child development surveillance. In recent decades, the focus of child stimulation transcends actions centered solely on children, expanding to the construction of a support network for development, as a poorly stimulating environment exposes them to risk factors and different developmental delays^(^ 10 ^)^. In addition, adverse childhood experiences will have consequences in the course of life, including parental experiences and trauma^(^ 11 ^)^, hence the importance of guiding families to care.

Undoubtedly, mothers are primarily responsible for providing direct care to their children^(^ 9 ^)^. Thus, health professionals need to support this mother so that stimulating acts, including playful and affective actions, especially for children at risk for development, may be understood by them as presupposed to the practice of care, including to enhance child development^(^ 12 ^)^, and thus try to reduce the negative effects of risk factors.

In this sense, the establishment of educational interventions with close relatives, namely mothers, about the evolution of development and the orientation regarding the use of auditory, visual, sensory, social and daily motor stimuli are fundamental strategies for the optimization of the development potential of the child, especially in the first 24 months^(^ 13 ^)^. Orienting the primary caregiver, in theory the mother, on aspects of development, valuing their empirical knowledge about child stimulation, will facilitate the construction of new opportunities for experimentation at home^(^ 14 ^)^.

Therefore, it is necessary to guide and exchange information with mothers about the correct use of activities that promote a healthy development. Communication, the fundamental axis of popular health education, is an essential aspect for them to learn to implement stimuli correctly and successfully, considering the characteristics and needs of the child^(^ 15 ^)^.

Based on the above, this study assumed that educational intervention is an effective strategy to improve maternal knowledge about promoting neuropsychomotor development in children under two years of age. Therefore, the following problematic question arose: Can educational intervention be considered an important instrument for the promotion of maternal knowledge in face of stimulating child development? The objective of this study was to analyze the effectiveness of an educational intervention with mothers of children under two years of age at risk for neuropsychomotor development.

## Method

Before-after intervention study with a quantitative approach, whose steps were guided by the *Quality Improvement Reporting Excellence* (SQUIRE) instrument. The research was developed in six Public Reference Centers in Early Childhood Education (CREIS) in rural and urban areas of a city located in Curimataú in Paraíba, Brazil. At the time of data collection, between March and June 2018, the municipality had six referral centers that assisted a population of 392 children between 0 (zero) months and 4 years, of which 137 were under two, which constituted a fundamental scenario for child development surveillance. Therefore, they provided a space favorable to conducting educational actions with relatives regarding the promotion of care, such as the stimulation of child development.

Initially, an assessment of neuropsychomotor development was carried out according to the manual of Child Development Surveillance in the Context of Integrated Management of Childhood Illness (IMCI)^(^ 16 ^)^ of the 137 children under two years attending the CREIS of the referred municipality. The study included only mothers of children at risk for development, whose children were enrolled and attending the city’s Reference Centers for Early Childhood Education during the data collection period. The justification for choosing children in this age group is based on the recommendation of the Ministry of Health^(^ 17 ^)^, which advocates it as a priority age group for the evaluation and identification of possible deviations of child development for the establishment of specialized treatment in a timely manner. In this sense, meeting the criteria mentioned, 52 mothers of children under two years participated in the data collection.

The mothers were invited to a meeting in the facilities of the Public Reference Centers in Early Childhood Education, and one of the authors introduced the research project and its stages, inviting them to participate. Agreement was given by signing the informed consent form. Then, a schedule was agreed upon according to the availability of mothers and researchers.

The first stage consisted of applying a data collection instrument from the pre-intervention perspective, which investigated maternal and child sociodemographic and biological characteristics. The second part of the instrument comprised 22 objective items, theoretically based on the Manual for Child Development Surveillance in the IMCI Context^(^ 16 ^)^, which inquired about maternal knowledge on child development and stimulation.

It is noteworthy that the data collection questionnaire was submitted to the evaluation of psychometric properties by 12 judges in the field of the study, which allowed to determine if the questions of the instrument measured what was intended, that is, met the criteria of clarity, precision and relevance. Although there is no consensus in literature regarding the ideal number of judges, we followed what literature recommended^(^ 18 ^)^, which suggests from 06 to 20 subjects. The choice of judges considered the *expertise* in child health issues, including people who developed research and had professional experience for more than two years in the area. Expert sampling was non-probabilistic by the snowball reference chain. The reliability of the questionnaire was verified by Cronbach’s alpha coefficient statistical tool, which ranged from 0.980 to 0.985, with no need for changes in its structure.

The educational workshops took place in March, one at each Reference Center for Early Childhood Education, with a workload of five hours each. In each workshop, on average, eight mothers participated. The content addressed the stimulation of child development, and the guidance given to mothers had as theoretical reference the manual Child Development Surveillance in the context of IMCI^(^ 16 ^)^.

The workshop started with a dynamic presentation. After this, the activities began, which were developed based on active methodologies, with problematization as the main teaching-learning strategy.

In this sense, anchored in the theoretical framework of popular education^(^ 19 ^-^ 20 ^)^, we sought to provide a space for dialogue in workshops. The intervention sought to instigate reflection in all mothers so that they could talk about their children’s child development. It also served as a space for listening, resignification and reinvention, based on the needs shown by the group. The problematization aimed at the exchange of experiences and construction of knowledge, based on the reality and challenges of each mother. Thus, based on what was already known and according to the knowledge gaps shown, a new knowledge shared by all was built. So, in this scenario, everyone learns and teaches. The triggering questions to stimulate debate and dialogue were: “What do you understand about child development?” and “What would you like to know about your child’s development and stimulation?”

During the educational interventions, discussions with the mothers were conducted, through dialogued exposure and the use of illustrative banners, regarding general aspects about child development, risk factors, the main developmental milestones and the stimuli that should be performed with children according to age groups.

Throughout the workshops, mothers were invited to handle dolls, using various materials such as mats, balls, towels, stimulation mats, toys, among others, to simulate practical tasks of visual pursuit, trunk control stimulation activities and different posture changes. During the workshop, mothers also built materials to stimulate their children, reported their own stories, experiences, challenges, ways of coping with problems, popular knowledge on the subject, their doubts, concerns and suggested that knowledge could be built jointly between the learner (mothers) and the educator (researchers).

It is noteworthy that an explanatory booklet with illustrations was presented and discussed with the mothers, elaborated by the researchers based on the Child Development Surveillance manual in the Context of IMCI^(^ 16 ^)^ and by a graphic design containing guidelines related to the exercises to be practiced at home, the concept of child development, risk factors and guidelines for performing child stimulation according to age groups up to 24 months.

After the completion of the educational interventions, a global evaluation of the workshop was carried out through the dynamic “How good… How sad… and How about…”, where the mothers were invited to complete the sentences with these terms, explaining their opinions about the educational session. After three months conducting the workshops, the second stage of data collection was performed, an opportunity in which the test was reapplied individually from the perspective of post-intervention, in order to reassess the maternal knowledge on the topic addressed.

Statistical analysis was performed using the *Statistical Package for Social Sciences (SPSS)*, version 21.0. Descriptive analysis of maternal sociodemographic characteristics (relative and absolute frequencies, mean, median, standard deviation) and inferential analysis for the pre and post-intervention experimental situation were performed, applying the nonparametric McNemar and Wilcoxon tests and the Rasch Model from Item Response Theory, which allows to determine the difficulty index of each question. It is noteworthy that, throughout this work, the statistical hypotheses were considered significant with the decision to reject the null hypothesis when the p value is ≤ 0.05, that is, the significance level to be considered will be 5%.

The study was approved by the Research Ethics Committee of the Health Sciences Center of the Federal University of Paraíba under protocol No. 82127817.3.0000.5188, according to the requirements established by Resolution 466/12 of the National Health Council that guides the practice of research with human beings.

## Results

All mothers, as shown in Table 1, agreed to participate in the study, totaling 52 with predominant age between 19 and 29 years (50.0%) followed by those with age between 30 and 39 years (48.1%). The interviewees reported stable marital union (75.0%) and education of eight or more years of schooling (57.7%). Most mothers lived in the urban area (51.9%) and had low socioeconomic status. Mostly, they performed no paid work activities (88.5%), since most of them were dedicated to domestic chores and child care, reducing the monthly *per capita* family incomethat was below one minimum wage (63.5%).

**Table 1:** Sociodemographic and biological characteristics of mothers and children who use Reference Centers in Early Childhood Education. Cuité, Paraíba, Brazil, 2018

Variables	n	%
Maternal age (years)		
19-29	26	50.0
30-39	25	48.1
>40	1	1.9
Marital Status		
Married/common-law marriage	39	75.0
Others	13	25.0
Paid work		
Yes	6	11.5
No	46	88.5
Family income		
< 1 minimum wage*	33	63.5
1 minimum wage	18	34.6
2 to 3 minimum wages	1	1.9
Schooling (years of study)		
Less than 1 year	2	3.9
1 to 3 years	6	11.5
4 to 7 years	14	26.9
8 years or more	30	57.7
Residence		
Urban area	27	51.9
Rural Area	25	48.1
Number of children		
1	35	67.3
2	14	26.9
3	3	5.8
Age of Child (Months)		
1 to 6 months	2	3.9
7 to 12 months	19	36.5
13 to 24 months	31	59.6
Child’s sex		
Female	27	51.9
Male	25	48.1
Total	52	100.0

* Minimum wage in Reais R$ 954.00 Brazil, 2018

Those who did paid work did so sporadically and received as day laborers. Most mothers had one child (67.3%), whose age ranged from 13 to 24 months (59.6%).

Table 2 shows the comparisons of the questions answered correctly by mothers about child development and stimulation, before and after the educational intervention. It was observed that there were statistically significant differences (p <0.05) in all items, except for question 4. Therefore, it is evident that the educational workshops promoted favorable changes in maternal knowledge about the content addressed.

**Table 2:** Number of maternal correct answers for questions about child development and stimulation before and after educational intervention. Cuité, Paraíba, Brazil, 2018

Questions	Educational intervention					
		Pre		Post		p-value*
		%	n	%	n	
The mother should encourage interaction with the child from birth with smiles and games, talking with the child during the bath and diaper changes.		8	15.4	47	90.4	< 0.001
A child at 2 months can smile when looking at the face of an adult.		13	25.0	48	92.3	< 0.001
A child at 3 months is able to roll alone.		14	26.9	48	92.3	< 0.001
The child begins to duplicate syllables around 3 months.		29	55.8	38	73.1	0.093
For a child to have a firmer neck from the age of 3 (three) months, they must be placed face down and a colored object should be used, in front of them, to draw their attention.		7 7	13.5	49	94.2	< 0.001
4-month-old children lying on their stomach can lift and hold their head firmly.		8	15.4	45	86.5	< 0.001
Children utters their first word at 4 months.		20	38.5	44	84.6	< 0.001
At 4 months, the child should be seated with support so that they can exercise their head, making it firmer.		8	15.4	38	73.1	< 0.001
Objects and toys that encourage fitting functions should be offered to the child from 5 months.		12	23.1	38	73.1	< 0.001
Breastfeeding helps in the development of the child, so it should be practiced exclusively, without other fluids and foods, until 6 months.		18	34.6	47	90.4	< 0.001
A child sits on the floor without falling at 7 months.		16	30.8	46	88.5	< 0.001
A child at 7 months can reach for objects.		14	26.9	27	51.9	0.026
Music helps to develop the child’s hearing and creativity and should be used from the age of 8 months.		13	25.0	51	98.1	< 0.001
From 9 months, the child should be encouraged to play with paper and crayons to scribble.		11	21.2	41	78.8	< 0.001
The child should be encouraged to build cube towers at 11 months of age.		12	23.1	42	80.8	< 0.001
The child can walk with support at 12 months.		11	21.2	51	98.1	< 0.001
Most children at 12 months can pick up small objects using tweezer movement.		12	23.1	42	80.8	< 0.001
Affection and love are beneficial feelings for the development of children and must be demonstrated from 12 months of age.		27	51.9	50	96.2	< 0.001
From 12 months onwards, toys should be offered in front of the child so that they can try to drag and crawl.		15	28.8	37	71.2	< 0.001
The child should be encouraged to play kicking a ball from 12 months.		9	17.3	46	88.5	< 0.001
A child should be encouraged to locate pictures of books and magazines from 18 months of age.		11	21.2	50	96.2	< 0.001
Most children at 24 months are able to remove some garment with adult help.		12	23.1	50	96.2	< 0.001
Total		300	26.2	975	85.2	< 0.001

* P-value = McNemar Test

According to the data shown in Table 3, there is a significant difference in maternal information regarding the general aspects of child development and stimulation before and after the educational intervention, that is, we observed an increase in the scores of correct answers expressed by the descriptive measures mean, median and standard deviation, confirming the effectiveness of the workshops developed in day care centers. Through the item response theory with the Racsh Model, a change was verified in the maternal difficulty index in answering each question of the instrument before and after the educational session. In this case, there is a reduction in the means and medians after the educational sessions, which reflects the mothers’ lower degree of difficulty in answering the questions at this moment when compared to the pre-intervention period.

**Table 3:** Comparison between maternal knowledge about child development and stimulation and difficulty in answering questions before and after educational intervention. Cuité, Paraíba, Brazil, 2018

Variable	Intervention	Mean	Standard Deviation	Median	p-value*
Maternal knowledge	Pre	5.77	1.85	6	< 0.001
	Post	18.60	1.94	19	
Degree of difficulty of maternal knowledge when answering the questionnaire	Pre	1.17	0.57	1.26	< 0.001†
	Post	-1.98	1.63	-2.18	

* P value = Wilcoxon test;

† Item Response Theory with Racsh Model

## Discussion

This research focused on the effectiveness of an educational intervention, performed with mothers, inherent to the stimulation of children under two years old at risk for developmental changes. Evaluation through tests applied before and after the workshop was an important strategy for estimating the effectiveness of the educational practice. The pre-test implies the recognition of the participants’ previous knowledge regarding the topic addressed, while results obtained after the intervention test reveal the improvement or not of the mothers’ level of knowledge^(^ 21 ^)^.

When analyzing the results of each item used to measure maternal knowledge about the stimulation of child development, it is observed that the proposed intervention was effective, considering that, after its completion, there was a significant increase in the correct responses of the questions answered by the mothers related to the development and changes in attitudes towards child stimulation, except for the fourth question, which identified insufficient maternal knowledge related to the assessment of language development in children.

In addition, the increase in knowledge coincided with the test phase in the post intervention perspective through the difference revealed by the reduction in the degree of difficulty in responding to the instrument used before and after the educational workshop. These results are consistent with studies conducted in Peru^(^ 22 ^)^ and Brazil^(^ 23 ^)^ with children at risk for development, which found effectiveness in intervention protocols based on guidance related to early stimulation transmitted to mothers to promote healthy development.

In this research, among the items that showed improvement of maternal knowledge, we highlight the stimulation of affective interaction, because mothers recognize the importance of love, affection and attention to the child as decisive aspects for a successful development. This reinforces the idea that affectivity is considered one of the fundamental care practices for the child, and the family should be stimulated in this regard, so that it can transform the reality in which the child lives^(^ 24 ^)^.

In addition to the affective aspect, the results also show evolution in maternal learning after participation in the workshop regarding the forms of stimulation of motor and sensory domains according to the age of the child. The use of problem-solving methodology in the workshops, coupled with the maternal practical simulation with puppets and the use of educational materials such as an illustrated booklet, may have been facilitating mechanisms to improve post-test knowledge in the predominance of the issues addressed.

This hypothesis corroborates a study that used educational materials to guide parents and relatives on child stimulation in order to improve or reverse motor development delays^(^ 25 ^)^. However, it should be emphasized that the educational materials must have in their purpose objectivity and accessible language on the theme addressed, because the lower the level of education of the individual, the clearer its content should be, otherwise, when poorly designed, these resources may also make it difficult for the user to understand^(^ 11 ^-^ 12 ^)^.

Similarly to intervention studies^(^ 22 ^-^ 23 ^)^, the mothers who are part of this research were instructed as to the positions, sensorimotor and affective stimuli that should be practiced according to the needs and age range of the child. However, considering the precepts of popular education in which learning is made from empiricism, we sought, through active listening, to previously value the maternal experiences regarding the forms of stimulation used daily, so that later the knowledge and attitudes towards children would be (re)constructed.

However, despite the promising results after the workshops, we recognized the identification of fragility of maternal knowledge regarding child communication. On this issue, it is conjectured that the lack of professionals trained in the area, such as the speech therapist, may compromise the promotion of educational health actions with parents regarding the guidance on stimulation and identification of possible changes in communication in children, reducing parents’ knowledge of the subject.

In this sense, it is essential to continue the educational strategies used in the workshops so that mothers can experience other moments of exchange of experiences and mutual learning, not only of content that was little learned, but also about various aspects of development. Thus, childcare skills are strengthened, as stimulation at home is also integrated with this practice.

It is believed that knowing the maternal experiences to later redirect the orientations according to the family reality was a fundamental aspect to obtain positive results in the teaching-learning process of the research on screen.

According to the principles of popular education, only through dialogue, the subjects, whether educators or learners, bring in their content the notion of knowledge from the perspective of participation and social transformation^(^ 26 ^)^.

Collective actions involving educational practices in health, such as those carried out in this study, need to be seen as moments of sharing ideas that respect diversity, being the health professional a true facilitator and a link between the community for the promotion of child development^(^ 27 ^)^.

In this sense, it is understood that, the presence of those who help facilitate their development properly is as important as the structuring and organization of the physical space for the child to develop^(^ 15 ^)^. Therefore, it is necessary to value the educational approach offered by the health professional to parents and family^(^ 28 ^)^.

The data shown strengthen the idea that teaching is not established by mere knowledge transfer, but by active listening and creation of possibilities that facilitate its construction based on pre-existing knowledge^(^ 26 ^)^.

It is conjectured, however, that the substitution of the traditional model of education based on a banking conception through which relations are verticalized and established by the transfer of information to the student, may have been a fundamental strategy to obtain satisfactory results in the educational post-intervention evaluation.

The number of correct answers after the educational intervention is considered to be of fundamental relevance to foster popular education and its methodology as an indispensable strategy for reorienting health practices^(^ 29 ^)^. In this context, it is necessary to reflect and plan despite the type of approach that will be used during educational interventions, as each group may have specificities that require different interventions, but direct contact has the potential to make knowledge more effective.

This is what was observed in an Iranian study^(^ 30 ^)^ portrayed with caregivers of children under the age of three, which found that one of the preferred educational approaches by caregivers to improve their knowledge on stimulating their development was face-to-face contact.

Although it is not possible to say with certainty what fostered the current maternal knowledge, considering that this was not the object of the proposed study, it is believed that factors such as the use of problematizing methodology in the workshops, added to the establishment of a horizontal relationship between the participants, ensuring the democratic, dialogic and participatory space, may have contributed to the success of the intervention.

Likewise, it is necessary to highlight that sociodemographic variables, such as maternal age, education, family income, number of children and lack of paid work outside home, have been considered influential in the evolution of knowledge or change in maternal behavior to perform appropriate health practices. According to a study, mothers over the age of twenty and with more than five years of study had a greater capacity for emotional involvement and stimulation, as well as a better ability to organize their home environment to stimulate their children^(^ 31 ^)^.

However, it is noteworthy that, although high education is considered an important aspect for improving maternal knowledge^(^ 14 ^)^, the data from this research project reveal that mothers with low educational level are also able to improve their knowledge about child development and learn to properly stimulate children.

Family income, according to a research conducted in Pakistan, also seems to influence the quality of the physical environment and, consequently, the quality of child development stimulation^(^ 32 ^)^. This is also what another study revealed, in which families with lower purchasing power had low rates for offering stimuli to children in the gross and fine motor dimensions^(^ 33 ^)^.

However, contradicting the findings mentioned, although family income is an important aspect for the supply of toys, this does not guarantee that a greater amount of material resources provides an adequate home environment, as revealed by research developed with children whose average age was 42 months. In this study, it was found that a better social class is not a sufficient condition to structure a home environment that provides opportunities for children’s motor performance in basic tasks^(^ 34 ^-^ 35 ^)^. These data corroborate the importance of providing educational actions that offer adequate guidance to families regarding the implementation of the right stimulus for development.

The participants of this research, despite being part of a less favored social class, revealed an improvement in their knowledge about the types of stimuli to be performed on the child and were interested in building possibilities and implementing them on a daily basis at home.

Presenting and discussing with mothers strategies to stimulate their children with household and recyclable materials was one of the approaches used in the educational workshops that possibly contributed to the practical viability of stimulation with their children at home, considering that most did not have favorable economic conditions to buy toys.

The proposal developed in this research based on popular education provided a sense of responsibility for the exchange of experiences and for the ability of participants to reflect on their reality and become active subjects for the transformation of their child’s microsystem, i.e. the home environment.

In addition to the active methodology, the mothers’ initiative to be present in the educational workshops may also have contributed to the positive results, since, when they became aware of the workshops, they showed interest in participating and a desire to improve their knowledge about the theme addressed. This aspect is very relevant, because the change of attitude is only possible from the personal will of each individual to learn something new related to previous knowledge, because otherwise all learned content will be quickly forgotten^(^ 27 ^)^.

Even in face of findings on the scientific contributions to the results obtained in this research, limitations need to be listed. Firstly, regarding the scope of the study that was restricted to the municipality, weakening the generalization of the obtained results. However, an overview of the region regarding the effectiveness of the intervention was obtained, with results similar to both national and international studies. Secondly, the limited number of participants and lastly, because it was a cross-sectional study that did not allow inferences of chance. Finally, given the relevance of the booklet to the knowledge of mothers of children at risk for development, we intend to validate it for future publication.

In this sense, it is suggested that works be developed with larger sampling. In addition, we suggest that observational studies be developed with the purpose of evaluating the family context and other contexts, in addition to the Public Reference Centers for Early Childhood Education, in order to verify more specifically the offer of stimuli in this microsystem for child development.

## Conclusion

This educational intervention proved to be effective because it improved the mothers’ knowledge on the stimulation of child development, contributing to the possibility of home practices with their children.

From the promising results obtained in this research, the use of problematizing methodology with the active participation of mothers in the learning process is recommended. In addition, valuing prior knowledge linked to the use of educational materials for learning at home also proved to be an important strategy for improving mothers’ knowledge.

The inclusion of actions that promote the stimulation of child development in the scope of primary health care and in Public Reference Centers in Early Childhood Education is suggested, in order to help parents and caregivers to apply appropriate stimuli for the promotion of child development in the home context.
